# Accuracy of neutrophil to lymphocyte and monocyte to lymphocyte ratios as new inflammatory markers in acute coronary syndrome

**DOI:** 10.1186/s12872-021-02236-7

**Published:** 2021-09-07

**Authors:** Ahmed Mohammed Shumilah, Arwa Mohammed Othman, Anwar Kasim Al-Madhagi

**Affiliations:** Microbiology and Immunology Department, Faculty of Medicine and Health Sciences, Sana’a University, Sana’a, Yemen

**Keywords:** Acute coronary syndrome, Monocyte to lymphocyte ratio, Neutrophil to lymphocyte ratio, Yemen

## Abstract

**Background:**

Inflammation plays a key role in the development of atherosclerosis and in the pathogenesis of acute coronary syndrome (ACS). Leukocytes and leukocytes ratios were recognized as inflammatory markers in predicting the presence and severity of ACS.

**Methods:**

This study aimed to investigate the diagnostic accuracy of neutrophil to lymphocyte ratio (NLR) and monocyte to lymphocyte ratio (MLR) with ACS. One hundred patients admitted to the Cardiac Center who were confirmed to have ACS and 100 healthy controls confirmed not to have ACS were enrolled in this study. ECG and troponin I test were used as gold standards to make sure that the participants with or without ACS. Total white blood cells (WBCs) count, NLR, and MLR values were estimated.

**Results:**

Total WBCs, neutrophil, and monocyte counts were significantly higher while lymphocyte counts were significantly lower in ACS patients than in the healthy controls (*p* < 0.001). NLR and MLR were significantly higher in ACS patients than in the healthy controls (*p* < 0.001). Among all the studied markers, NLR was found to be the strongest predictive marker of ACS (OR: 3.3, *p* < 0.001), whereas MLR was non-significant (*p* > 0.05). A cut-off value of 2.9 of NLR had 90% sensitivity and 88% specificity while 0.375 cut-off value of MLR had 79% sensitivity, 91% specificity for predicting ACS presence.

**Conclusions:**

NLR is a simple, widely available, and inexpensive inflammatory marker which can be an auxiliary biomarker in the diagnosis of ACS with a cut-off value of 2.9 in our population.

## Background

Cardiovascular diseases (CVD) remain the major cause of death worldwide. Atherosclerosis disease, the underlying process that results in ACS, is responsible for a large rate of CVD. In 2016, out of the 17.7 million cardiovascular deaths, about 85% were due to atherosclerosis disease [1]. Inflammation plays a key role in the development of atherosclerosis and in the pathogenesis of ACS. To show this inflammation, numerous markers such as hs-CRP, fibrinogen, and interleukins have been used used [2, 3]. Leukocytes are major mediators of inflammation. Therefore, leukocytes and its subtypes were studied as an inflammatory marker in predicting adverse events in ACS patients [4–6]. The systemic immune-inflammation index was independent predictor of the no-reflow phenomenon in patients with ACS [7].

Recently, NLR, MLR, and platelet to lymphocyte ratio (PLR) have emerged as one of the most important novels widely available, inexpensive, and robust inflammatory markers which can aid in the prognosis and diagnosis of ACS [3, 8–13].

Diagnosis of ACS is mainly based on the troponin, electrocardiograph (ECG), and other cardiovascular imaging modalities; however, these investigations are expensive and time-consuming. Discovering simple markers become important in order to help diagnostic accuracy and to provide prognostic information about this disease.

Since it has been hypothesized that the NLR and MLR reflect ongoing inflammation in ACS, this study aimed to investigate the diagnostic accuracy of NLR and MLR with ACS.

## Methods

### Study design and definition

This study was testing a test case–control study carried out at Al-Thawra General Hospital in Sana'a city from April 2019 to July 2020. It was conducted on 100 patients who admitted to the Cardiac Center and were confirmed to have ACS (patients group) and 100 healthy individuals (control group).

ECG and troponin I test were used as gold standards to make sure that the participants with or without ACS. The ACS group included ST elevated myocardial infarction (STEMI), non-ST elevated myocardial infarction (NSTEMI), and unstable angina (UA) that were defined based on the criteria formulated by updated guidelines [14]. The control group was selected and defined as healthy individuals matching with the patient group for age and sex and confirmed not to have ACS by the gold standard tests. Participants with hypertension and diabetes and other metabolic CV risk factors were excluded from the control group.

All male and female patients who diagnosed to have ACS and their age equal to 18 years old or over, and ACS patients with or without hypertension were included in the study. Patients with CVD other than ACS, ACS patients aged lower than 18 years, ACS patients with diabetes mellitus, medical conditions, or treatments that are known to affect the WBCs count were excluded from this study. Diabetes and hypertension were defined based on the criteria formulated by updated guidelines [15]*.* The study was approved by the local Ethics committee at Sana’a University and informed consent of the patients and controls.

### Laboratory analysis

EDTA-venous blood sample was collected from each patient and healthy control at admission and was analyzed at the same time. Total WBCs, differential WBC count, mean platelet volume (MPV), and red cell distribution width (RDW) were measured by using an automated analyzer (Mindray Medical International Limited, Shenzhen, China). NLR and MLR were calculated from differential WBC count as a ratio of neutrophil and monocyte cell counts to lymphocyte cell counts. The PLR was calculated as the ratio of platelets cells count to lymphocyte cells count. Erythrocytes sedimentation rate (ESR) was performed by the Westergren method. Body mass index was calculated by taking the body weight in kilograms (kg) and dividing it by the height in meters (m) squared. Hypertension was evaluated by using a simple mercury sphygmomanometer to measure systolic and diastolic blood pressure.

The ECG investigation was conducted and read by a cardiologist at the Heart Center of Al-Thawra General Hospital, where we focused on the following; T-wave, Q waves, and ST-segment. Cardiac troponin I was detected in serum by qualitative, membrane-based immune-assay (Cardiac Troponin I Rapid Test Cassette). Biochemical measurements such as CK-MB, CK, and glucose, were obtained from the patient's files.

### Statistical analysis

For comparing two groups, a Student-t-test or Mann–Whitney U test was used for numerical variables while the Chi-square test was used for categorical variables. A correlation between the numerical variables was determined by using Pearson’s or Spearman correlation test. The strength of an association of the markers with the disease was assessed using logistic regression for the parameters showing a significant association with ACS disease on univariate analysis (*p* < 0.05). Receiver operating characteristic (ROC) curve analysis was performed to determine the accuracy of NLR and MLR for predicting ACS presence**.**

## Results

The study population comprised 200 participants, 100 apparently ACS (60 males and 40 females), and 100 healthy controls (60 males and 40 females). The ages and genders were matched between the two groups.

Total WBCs, neutrophil, and monocytes counts, were significantly higher while lymphocytes count was significantly lower in ACS patients than in the healthy controls (*p* < 0.001). NLR and MLR were significantly higher in the ACS patients than in the healthy controls (*p* < 0.001). Raised levels of PLR, MPV, ESR, and RDW were also found in ACS patients as compared to healthy controls (*p* < 0.05). The hematocrit was lower in ACS patients than in the healthy controls. The frequency of smoking and the mean BMI were significantly higher in the ACS patients than in the control group (*p* < 0.05) (Table 1).

**Table 1:** Comparison of all variables between ACS patients and controls groups

Variables	Patients (n = 100)	Controls (n = 100)	*p* Value
Age (years)	55.5 ± 15	54.1± 15	0.516
Genders (males) (%)	60	60	1.00
Smokers (%)	62	45	0.023
BMI (kg/m^2^)	23.9 ± 3.5	21.4 ± 3.1	<0.001
WBC × 10^3^/μL	9.6 ± 2.5	6.8 ± 1.9	< 0.001
Neutrophil × 10^3^/μL	7.2 ± 2.4	3.8 ± 1.5	< 0.001
Monocyte × 10^3^/μL	0.69 ± 0.28	0.47± 0.22	< 0.001
Lymphocyte × 10^3^/ μL	1.3 ± 0.50	2.1 ± 0.62	< 0.001
Platelets × 10^3^/μL	249 ± 81	263 ± 52	0.152
NLR	6.5 ± 3.0	1.9 ± 0.9	< 0.001
MLR	0.61 ± 0.29	0.23± 0.10	< 0.001
PLR	211 ± 74	131 ± 42	< 0.001
MPV/fl	9.8 (8.6)	9.6 (5.9)	0.003
ESR (mm/h)	47 ± 23	17 ± 9	< 0.001
RDW (%)	14.8 (12.5)	13.4 (14.6)	< 0.001
Hematocrit (%)	37.5± 6.7	41.9 ±5.9	< 0.001

*ES R* erythrocyte sedimentation rate, *MLR* monocytes to lymphocyte ratio, *MPV* mean platelet volume, *NLR* neutrophil to lymphocyte ratio, *PLR* platelet to lymphocyte ratio, *RDW* red cell distribution width, and *WBC* white blood cell count

### Correlation of NLR and MLR with inflammatory and myocardial infraction markers

The correlation analysis observed that NLR and MLR markers were positively correlated with each other, inflammatory cells, and myocardial infarction (MI) markers. Although the correlation was moderate with inflammatory markers and weak with MI markers, it was statistically significant (*p* < 0.05) (Table 2).

**Table 2:** Correlation of NLR and MLR with inflammatory and MI markers among ACS patients

Markers	NLR (95% CI)	*p* Value	MLR (95% CI)	*p* Value
NLR	1	< 0.001^a^	0.616 (0.493–0.715)	< 0.001^a^
PLR	0.536 (0.385–0.649)	< 0.001^a^	0.427 (0.209–0.555)	< 0.001^a^
WBC	0.464 (0.249–0.655)	< 0.001^a^	0.205 (0.019–0.442)	0.041^a^
Neutrophil	0.622	< 0.001^a^	0.234	0.019^a^
Lymphocytes	− 0.774	< 0.001^a^	− 0.647	< 0.001^a^
ESR	0.106	0.296^a^	0.222	0.027^a^
CK-MB	0.341 (0.135–0.565)	0.001^b^	0.307 (0.104–0.493)	0.003^b^
CK	0.280 (0.033–0.512)	0.032^b^	0.350 (0.059–0.554)	0.007^b^

*CK* creatinine kinase, and *CK-MB* creatinine kinase-myoglobin binding

^a^Pearson, ^b^Spearman’s

### NLR is the independent predictive marker for the presence of ACS

The strength of association of the NLR and MLR markers with ACS was assessed by multivariate regression analysis and is presented in Table 3. After adjustment for covariates include WBCs, NLR, MLR, MPV, RDW, ESR, PLR, Hematocrit, and smoking in multivariate regression, NLR was found to be the strongest predictive marker for ACS (OR: 3.34; *p* = 0.014), followed by MPV, RDW, and ESR, whereas MLR and other markers were not significant (*p* > 0.05) (Table 3).

**Table 3:** Multivariate logistic regression analysis for the presence of ACS

Markers	S.E	*p* Value	Exp (B)	95% CI for exp (B)	
				Lower	Upper
NLR	0.490	0.014	3.340	1.279	8.722
MPV	0.327	0.006	2.455	1.292	4.664
RDW	0.160	0.040	1.39	1.016	1.901
ESR	0.047	< 0.001	1.197	1.090	1.313
MLR	2.679	0.597	4.12		
PLR	0.010	0.313	1.01		
WBC	0.314	0.938	0.976		
Hematocrit	0.066	0.125	1.106		
Smoking	0.740	0.827	1.175		
Constant	8.311	0.001	0.000		

*Exp* (*B*) exponentiation of the coefficients/odds ratios of the predictive markers, *CI* confidence interval, *S.E* sample error

### The accuracy of NLR in detecting ACS

**Fig. 1:**
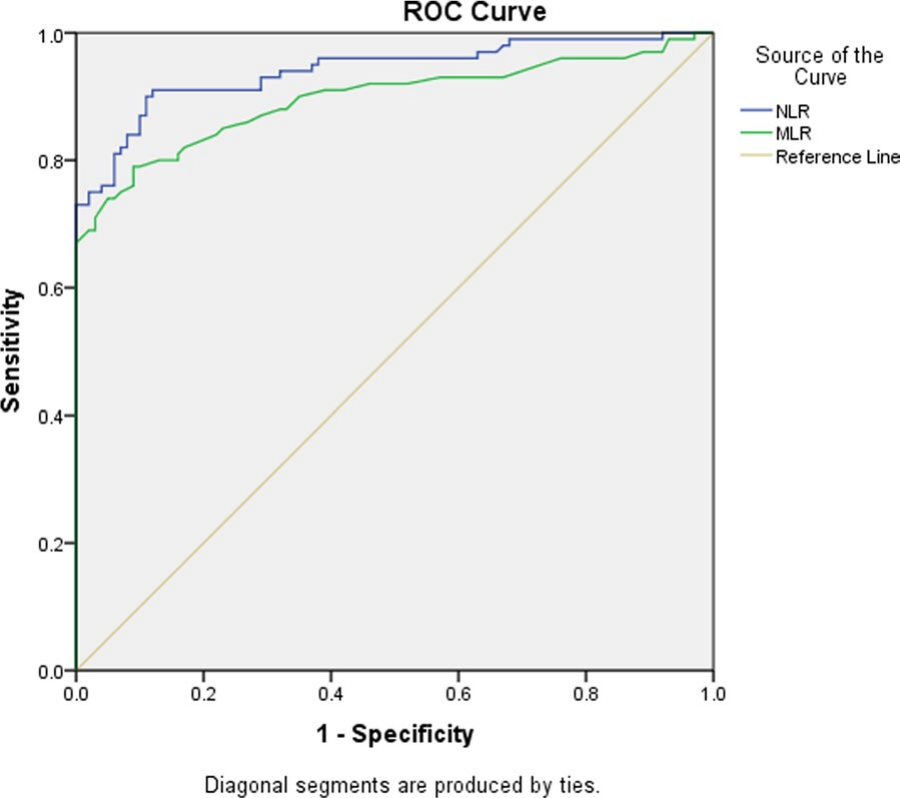
Receiver operating characteristic (ROC) analyzes of NLR and MLR for diagnosis of ACS

The diagnostic accuracy of the NLR and MLR markers for the diagnosis of ACS was investigated by ROC analysis Fig. 1. Based on ROC curve, NLR marker exhibited a higher area under curve (AUC: 0.941, *p* < 0.001), followed by MLR (AUC: 0.896, *p* < 0.001). The suitable cut-off value of NLR for the diagnosis of ACS was found to be 2.95 with 90% sensitivity, 88% specificity, 89.7% negative predictive value (NPV), and 88.2% positive predictive value (PPV). The suitable cut-off value of MLR for the diagnosis of ACS was found to be 0.375 with 79% sensitivity, 91% specificity 81.2% NPV and 89.7% PPV (Fig. 1).

## Discussion

To the best of our knowledge, this is the first study aimed to investigate the diagnostic accuracy of NLR and MLR in detecting the presence of ACS in Yemen. Both groups studied aged 55/54 ± 15 year, therefore a share is represented by elderly subjects. Furthermore, the distribution between the two genders in this study is not uniform (60% vs. 40%). As observed in the REPOSI study, coronary artery diseases were more common among males than females and the risk factors for heart diseases such as diabetes, chronic kidney disease and chronic obstructive pulmonary disease were also more frequent in males than in females [16, 17].

We herewith demonstrate that total WBCs, neutrophil, and monocytes counts were higher while lymphocyte was lower in ACS patients than in healthy controls. This increase of total WBCs, neutrophil, and monocytes counts in ACS patients may be due to the inflammatory response during atherosclerosis and a probable cause of lymphopenia include decreased production as a result of increased steroid level due to stress and increased apoptosis triggered by increased inflammation thereby resulting in elevated NLR and MLR markers [18, 19].

NLR is an integrated reflection of two dissimilar yet balancing immune pathways and is a better predictive of inflammation in ACS than neutrophils or lymphocytes alone [20, 21]. In our study, we demonstrate that ACS patients had significantly higher NLR and MLR compared to healthy controls. This difference is in agreement with previous studies [10, 22, 23]. Other studies also reported that NLR was significantly higher in the troponin-positive than in the troponin-negative patients [8, 9, 24].

NLR and MLR markers were positively correlated with inflammatory cells and MI markers including CK and CK-MB in ACS patients. This may indicate that these markers may reflect inflammatory response and myocardial injury during atherosclerosis. These findings have been described by several other studies [10, 11, 21, 25, 26]. MLR was positively correlated with ESR while the NLR was not significant. A recent study has documented that adiponectin plays a key role in the progression of coronary plaques [27].

The sensitivity of NLR in our study is relatively similar to those reported by Yilmaz et al. [4] and Corriere et al. [28] who found that NLR has 93% and 97% sensitivity for predicting the presence of thrombus and carotid plaques respectively. Their results support those in our study that stated that NLR has high sensitivity to reflect ongoing inflammation associated with ACS. Thus, we suggest that NLR may be served as a valuable biomarker for the diagnosis of ACS.

Among all studied markers, NLR was found to be the strongest independent marker of ACS presence. This result is in agreement with two other studies which reported that NLR was a strong independent predictor of ACS in chest pain patients [29, 30]. Other studies also reported that NLR independently predicted troponin positive in chest pain patients [9, 24]. However, we achieved a greater degree of accuracy (AUC-0.941; *p* < 0.001; sensitivity: 90%; specificity: 88%) as compared to those reported by previous studies. This is because those studies are different from our study in two main aspects: Firstly, unlike the current study, the control groups were not healthy individuals but included patients with chest pain where the inflammatory response is expected to be absent or less prominent in healthy compared to patients with chest pain. This explains why the specificity of NLR was higher in the current study compared to previous studies. Secondly, the higher frequency of STEMI sub-type among ACS group in our study compared to the previous studies where the inflammatory response is expected to be more prominent in STEMI patients compared to those with NSTEMI/UA [31]. This explains why the sensitivity of NLR was higher in the current study compared to those studies.

Compared to NLR, MLR was a non-significant marker for ACS (*p* > 0.05) and has a lower accuracy for the diagnosis of ACS. There is no previous study so far that discussed the predictive value of MLR in the discrimination of ACS patients, but there is only a study that has been conducted on patients with stable angina and showed that MLR was significantly independent predictor of a thin cap fibrous atheroma, with a sensitivity of 73.7% and a specificity of 61.8% [32].

In addition to NLR marker, the MPV, RDW, and ESR were also significantly independent predictive markers of ACS. These results were consistent with results in previous studies [33–36]. Although these studies differ in some methodologies from our study, they support our findings that MPV and RDW were independent predictive markers of ACS in different populations.

The current study revealed that MPV, PLR and RDW values were significantly higher while hematocrit was significantly lower in ACS patients than in the healthy controls (*p* < 0.001). This hematological index has been demonstrated to be useful to evaluate inflammatory status, assess platelet activity, and predict adverse outcomes in patients with ACS [10, 37–42]

There are several studies in the literature designed with the purpose of determining the accuracy value of NLR for the diagnosis of ACS [4, 9, 24, 28–30]. Our study is distinct from previous studies in that the individuals in the control group were healthy. In addition, the ACS patients and the control group were matched for gender and age. These variations need to be considered while using NLR for proposing the diagnosis of ACS [19]. Therefore, we achieved a greater degree of accuracy as compared to previous studies.

Hyperglycemia and insulin resistance may influence MI development by inducing oxidative stress, endothelial dysfunction, and vascular inflammation. Suggesting that ACS patients with diabetes may have a higher vascular inflammatory response compared to ACS without diabetes [43, 44]. Despite exclusion of diabetic patients from our study, blood glucose level was not measured to determine stress hyperglycemia during ACS thus determinations of NLR, MLR and blood glucose are recommended to be considered in future studies.

NLR is a simple, inexpensive, widely available, and robust inflammatory marker. Therefore, we believe that the NLR will provide initial information, to clinicians' decisions, especially in small centers, to determine the need for further imaging modalities in the assessment of ACS at its earlier stages.

### Study limitations

The present study must be interpreted within the context of its potential limitations.

- First, this was a single-center designed study and the sample size was too small, hence further large comparative multi-center studies will be needed.
- Second, the patients in the STEMI sub-group were higher compared with the NSTEMI/UA subgroups thus equal sub-groups of ACS are recommended to be enrolled in future studies.
- Third, we could not compare NLR and MLR with inflammatory markers (e.g. Hs-CRP) and pro-inflammatory cytokines (e.g. interleukin-6), because they were not evaluated in our study or routinely assessed in our study population.
- Fourth, we could not mesure the effect of stress hyperglycemia on NLR and MLR markers.

## Conclusions

The results of this study revealed that NLR was the strongest predictive marker of ACS, so the study recommends using NLR as a simple, inexpensive, and widely available inflammatory marker which can be an auxiliary biomarker in the diagnosis of ACS. Also, further large-scale and comprehensive studies are highly recommended.

## Data Availability

The datasets used and analyzed during the current study are available from the corresponding author on reasonable request.

## Abbreviations

ACS: Acute coronary syndrome
BMI: Body mass index
CHD: Coronary heart disease
CK: Creatine kinase
CK-MB: Creatine kinase myoglobin-binding
CVD: Cardiovascular diseases
ECG: Electrocardiogram
ESR: Erythrocyte sedimentation rate
Hs-CRP: High-sensitivity C-reactive protein
MI: Myocardial infarction
MLR: Monocyte to lymphocyte
MPV: Mean platelet volume
NLR: Neutrophil to lymphocyte ratio
NSTSEMI: Non-ST-segment elevation myocardial infarction
PLR: Platelet to lymphocyte ratio
RDW: Red cell distribution width
STSEMI: ST-segment elevation myocardial infarction
UA: Unstable angina
WBC: Total white blood cells
WHO: World health organization
